# Sprained Ankle—II

**Published:** 1909-07-17

**Authors:** 


### Orthopædics.

### SPRAINED ANKLE—II.

The treatment of a simple sprained ankle, when once a more serious lesion has been definitely excluded, is mainly directed towards improving the local reaction which is Nature's own superior way of dealing with the condition. The orthodox method of treatment was, and in many quarters still is, to prescribe complete rest, with cold lotions applied over the part. "A wet handkerchief, a bowl of cold water, a sofa, plenty of light literature, and a well-aired room"—this summed up the essentials of treatment as many understood it. No doubt this line of treatment has its good points. It is true the cold water compresses do not assuage the pain of the sprained ligaments so quickly and completely as do hot boracic lotion poultices, while complete rest again is by no means advisable in every case. The simplest cases got well without any bad result under this treatment; but they would have got well equally quickly if left to Nature herself without any interference from the practitioner. The more severe cases, on the contrary, did not do so well. The writer has had occasion to examine a series of patients who had had severe ankle sprains some years ago, and who had been treated on the usual lines "with complete success." On examination, in nearly every case some degree of flat-foot or some difference in the originally injured foot was observable, directly traceable, certainly in the majority of cases, to the original injury.

We have long held it as an axiomatic truth that complete immobility in the case of fractured bones is not conducive to quick and strong union. Cases are not unknown in which ambitious surgeons have so capably wired together a fractured long bone that no movement was possible between the apposed ends, with the result that no union occurred until the wires had been taken out, and the bone ends permitted to rub against each other. Excessive movement is, of course, incompatible with quick union, and apparently equally incompatible is excessive immobility. A slight degree of irritation is necessary to stimulate a tissue to growth. This we have learned by experience is the case in long bones, and later researches have demonstrated that it is equally the case with other tissues. A muscle, for example, that is subjected to slight movement repairs its rents much more permanently and much more securely than one which has been absolutely immobilised. This has been shown to be the case in wounds of the diaphragm, a muscle which is continually in action, and it is no doubt true of ligamentous tissues as well. It is therefore open to question whether complete rest is the best treatment in cases of sprain. Nor can it be held that passive movement in the shape of massage is an adequate substitute for the natural movements which occur at the seat of injury. Massage should be started much earlier than active movement, but should never be allowed, even from the first, to replace active movements made by the patient himself.

Having carefully examined the case and satisfied himself, as far as it is possible to do so, that the lesion is a simple one, uncomplicated by fracture or dislocation, the practitioner should treat it as such. A simple boracic lotion compress, put on moderately warm and changed every four hours, acts excellently in relieving pain and diminishing swelling. In an ordinary case this is all that is required in the way of applications, and in every case it is useful to commence the treatment with such compresses repeated three or four times. Rest from active exertion should be enjoyed, but passive and voluntary movements should be commenced at an early stage, and the patient must be encouraged to put in action the calf muscles by alternately flexing and extending the ankle. At first this series of movements must be very cautiously performed, and will probably give rise to some pain. It should be helped by gentle massage, the hand of the operator being anointed with an emollient cream. Emulsions and liniments in themselves do not materially hasten the cure of a simple sprain, but they are useful, in so far as they give the patient an impression that more is being done for him than is actually the case—a satisfactory delusion when the innate faith of the public in embrocations, liniments, and ointments is borne in mind. What undoubtedly does good is the massage, and this may be done as effectively with simple olive oil. This massage, together with the boracic fomentations, rapidly relieves the pain, which even in a simple sprain is often very severe, and in a week's time the patient is usually able to go about. Treatment by massage must, however, be persevered in till all stiffness and feeling of soreness about the site of the lesion have disappeared.

Where complications exist or are suspected, these must be the primary consideration. With the more serious, such as fracture and dislocation, it is unnecessary to deal here: their existence removes the case from the category of sprains. Superficial wounds and scratches, which are by no means unusual in sprain cases, should be treated by boracic fomentations if there is any suspicion of sepsis. This is especially advisable in the case of club patients and poor-class patients generally, who may be wearing cheaply-dyed stockings, the dye of which may act as an irritant, and in some cases may lead to much distress. Such superficial abrasions are troublesome, as they interfere with proper massage; where they are so extensive as to make rubbing an impossibility for the first few days, passive flexion and extension movements, and massage of the calf and shin muscles, must be commenced from the first. An unpleasant and distressing complication is tenosynovitis, generally of the plastic variety. This may affect the flexor or extensor tendons, but appears to be more frequent in the Achilles tendon and the peronei. Counter-irritation may be used to treat it, but the writer has never found this of much service. In any case, the plasters should be small—of the size of a threepenny bit—and applied not only over the actual site of the lesion, but a few inches higher up along the course of the tendon. Boracic fomentations, and in a few cases cold compresses, do more good in such cases of tenosynovitis than any embrocation or liniment, besides being much more comforting to the patient. One of the best methods of treatment, in every way worthy of a trial in private practice, is by means of a Bier's bandage. This is applied high up over the thigh, just below the groin, and left on for eight or more hours. It acts magically in some cases, all the pain, discomfort, and crepitation disappearing under the hyperæmia.

Later sequelæ should never be disregarded, and it is as well for the practitioner to examine his cases a month or six weeks after he has written them off as cured. The slightest tendency towards flat-foot should receive immediate attention, and a celluloid or cork inset, properly modelled to fit the arch of the foot, should be worn. Passive and active movements should be continued in such a case, but the ordinarily prescribed "foot" exercises which are supposed to "strengthen" the arch may be omitted, as they are hardly likely to benefit the case, and often do more harm than good. Other sequelæ, such as arthritis and neuropathic joint in a tabetic patient, can hardly be guarded against except by careful attention from the first time the case is seen. Immobility or limitation of movement, however, are preventable sequelæ, and should never follow an even bad sprain if the latter is properly treated.

